# Spotting Trends in Organocatalyzed and Other Organomediated (De)polymerizations and Polymer Functionalizations

**DOI:** 10.1002/anie.202203043

**Published:** 2022-08-10

**Authors:** María Valle, Marta Ximenis, Xabier Lopez de Pariza, Julian M. W. Chan, Haritz Sardon

**Affiliations:** ^1^ POLYMAT University of the Basque Country UPV/EHU Jose Mari Korta Center Avda Tolosa 72 20018 Donostia-San Sebastian Spain; ^2^ University of the Balearic Islands UIB Department of Chemistry Cra. Valldemossa, Km 7.5 07122 Palma de Mallorca Spain; ^3^ Institute of Sustainability for Chemicals Energy and Environment (ISCE2) Agency for Science Technology and Research (A*STAR) 1 Pesek Road, Jurong Island Singapore 627833 Singapore

**Keywords:** Depolymerization, Organocatalysis, Polymerization, Postpolymerization Functionalization, Transition-Metal-Free Catalysis

## Abstract

Organocatalysis has evolved into an effective complement to metal‐ or enzyme‐based catalysis in polymerization, polymer functionalization, and depolymerization. The ease of removal and greater sustainability of organocatalysts relative to transition‐metal‐based ones has spurred development in specialty applications, e.g., medical devices, drug delivery, optoelectronics. Despite this, the use of organocatalysis and other organomediated reactions in polymer chemistry is still rapidly developing, and we envisage their rapidly growing application in nascent areas such as controlled radical polymerization, additive manufacturing, and chemical recycling in the coming years. In this Review, we describe ten trending areas where we anticipate paradigm shifts resulting from novel organocatalysts and other transition‐metal‐free conditions. We highlight opportunities and challenges and detail how new discoveries could lead to previously inaccessible functional materials and a potentially circular plastics economy.

## Graphical abstract

The latest trends in polymer synthesis, depolymerization, and postpolymerization functionalization are reviewed, whereby organocatalysis and other organomediated methods take center stage. With today's growing emphasis on sustainability, we describe how transition‐metal‐free strategies offer fresh opportunities to access novel functional materials and also to valorize postconsumer plastic waste.

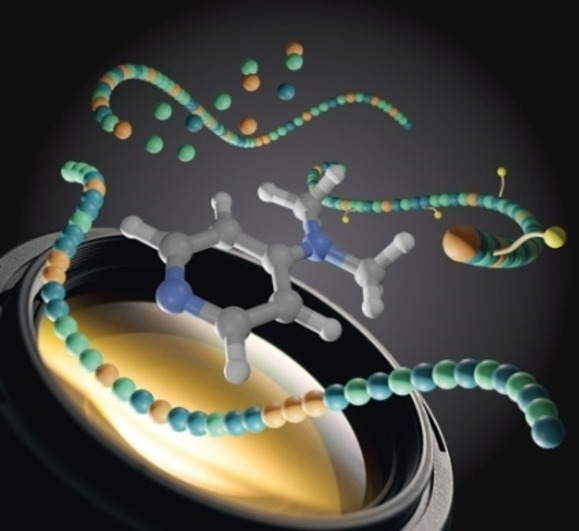

## 1 Introduction

Organocatalysis has now become a valuable tool for polymer chemists to mediate chemical reactions. It provides a robust alternative in promoting many polymerization reactions that lead to well‐defined polymers.^[1]^ Since the seminal work of Hedrick and Waymouth on the 4‐dimethylaminopyridine (DMAP)‐catalyzed ring‐opening polymerization (ROP) of lactide, the application of organocatalysts to polymerization processes (e.g., chain growth, step‐growth, controlled radical, or ring opening), has provided new strategies for catalytically activating functional monomers or chain‐ends and for controlling the polymerization under mild conditions. This has led to an explosion in the diversity of polymeric materials featuring a wide range of intriguing structural and functional macromolecular architectures.^[2]^ Several classes of organic activators (catalysts, initiators, or promoters), including Brønsted/Lewis bases and acids (e.g., amines, phosphines, or carbenes), and mono‐ or dual‐component systems, have been utilized to catalyze the polymerization of various synthons. In addition, they have also been utilized for plastic deconstructions in the context of circular economy of polymeric materials.^[3]^ For this reason, organocatalytic mediation is expected to play an important role in paving the way towards the recycling of plastics. The key motivation for transitioning from metal‐based catalysts to organocatalysts resides in their ability to exquisitely control the catalytic activity and selectivity of polymerizations, and in their potential separation and recovery from the final polymer.[^4^, ^5^, ^6^]

The initial development of organocatalysts was based on their relatively low toxicity and easier removal compared to their counterpart transition‐metal‐based catalysts,^[7]^ which boosted their development in biomedical, personal care, microelectronics, and packaging applications.[^8^, ^9^] Indeed, the mechanistic diversity of organocatalytic polymerizations has created new opportunities for polymerization and depolymerization reactions alike. Despite all these advances, the use of organocatalysis in (de)polymerizations is still a subject of intense interest as many potential features remain unexplored. We envisage that new emerging applications of organocatalysis in nascent areas such as controlled polymerization, additive manufacturing, and chemical recycling will enjoy widespread attention over the next 10 years and beyond. Therefore, for this Review, we have selected ten burgeoning areas in which we anticipate paradigm shifts stemming from the design of new organocatalysts. We have selected not only examples based on truly organocatalyzed systems but also transition‐metal‐free systems whereby organomediated transformations play an important role.

## 2 Burgeoning Areas in Organocatalyzed and Other Transition‐Metal‐Free Mediated Polymerizations, Polymer Functionalization, and Depolymerization

### 2.1 Organocatalyzed Photoredox Polymerization

Controlled living polymerization (CLP) is defined as a form of chain‐growth polymerization where the polymeric chains do not undergo termination reactions.^[10]^ Typically, a control agent mediates the process by activating/deactivating the polymer chain growth, thus allowing spatiotemporal control over the final macromolecule.

In this regard, atom transfer radical polymerization (ATRP) has dominated the field of CLP due to its robust and versatile nature and its ability to afford well‐defined polymers with precise molecular architectures.[^11^, ^12^] In early works, metals like Cu or Ru were used as catalysts for ATRP[^13^, ^14^] and later on, Ir was successfully used as a photoredox catalyst (PC), opening the field for light‐activated controlled polymerizations.^[15]^ In the past few years, organocatalyzed ATRP (O‐ATRP) has been achieved with organic compounds working as the PC. Miyake and Corbin have recently published a detailed review on O‐ATRP, evaluating the mechanistic insights of the process, key features for catalyst design, and possibilities for this CLP.^[16]^ For the purpose of this Review, we aim to emphasize the recent advances based on the structure–property relationship of the organocatalysis, which have a direct impact on the mediation performance.

**Figure 1:**
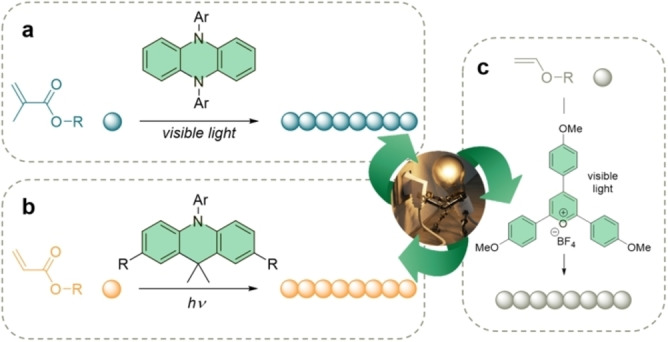
a) Example of visible‐light photoredox‐catalyzed O‐ATRP of methacrylates.^[21]^ b) First example of photoredox‐catalyzed O‐ATRP of acrylates using dihydroacrydines.^[29]^ c) Living cationic polymerization of vinyl ethers using pyrylium photocatalysts.^[30]^

Since the initial studies with perylene^[17]^ and phenothiazine^[18]^ in O‐ATRP, the design and development of innovative PCs, whose photophysical and chemical properties determine their performance, have been revealed. In Theriot and Miyake's work, by using ethyl α‐bromophenylacetate (EBPA) as an initiator, perylene was able to polymerize methyl methacrylate (MMA) under sunlight irradiation.^[17]^ This reaction was extended to other methacrylates and functionalized vinyl monomers, but low initiation efficiencies and relatively high dispersities (*Đ*) were observed. Nevertheless, it represented the first example where visible light was used to trigger polymerization. Phenothiazine derivatives, on the other hand, successfully polymerized methacrylates in a controlled manner under 380 nm UV light at room temperature, producing polymers with good *Đ* and relatively high molecular weights (MWs). It is noticeable that this catalyst was able to polymerize dimethylaminoethyl methacrylate, which may poison metal‐based catalysts as a coordination ligand.^[18]^ Expanding the phenothiazines scope, Nguyen and co‐workers achieved the PC synthesis of methacrylate block copolymers (BCPs) with controlled MWs and relatively narrow dispersities.^[19]^ Later on, Hu et al. applied the UV‐photocatalyzed O‐ATRP to continuous flow for the polymerization of MMA and the corresponding block‐copolymers with methacrylic acid and styrene in a controlled fashion.^[20]^ Further attempts on improving catalyst performance focused on achieving strongly reducing excited states for the PCs and a shift towards visible‐light absorption. Dihydrophenazines proved to exhibit both features and some progress has been reported.[^21^, ^22^, ^23^] Through computational design, the catalyst structure and the polymerization conditions have been optimized, achieving low *Đ* and tunable MWs upon activation/deactivation of the light source (Figure 1a). In parallel, the chemical modification of the phenoxazine family aimed for the same features. Miyake and McCarthy succeeded in the polymerization of MMA in a controlled fashion using core‐modified *N*‐arylphenoxazines in the presence of air.^[24]^ The structure–property relationship in the catalyst design was further explored by Son, Lee, et al., and this found application in the preparation of amphiphilic BCPs under sunlight irradiation.^[25]^ Aside from perylene, other polycyclic aromatic hydrocarbons (PAHs) have been explored in O‐ATRP. Anthracene and pyrene were explored by Yagci and co‐workers but the overall performance of those catalysts resulted in moderate to poor control over the polymer architecture and, especially for anthracene, radical side reactions of the catalyst were observed.^[26]^ Later on, highly conjugated electron‐rich thienothiophene derivatives offered greater control in the photocatalyzed O‐ATRP of MMA, also responding to on/off light experiments.^[27]^ Very recently, Liao and co‐workers successfully synthesized oxygen‐doped anthanthrene (ODA) and explored the effect of the heteroatom doping in the PAH's core. The O‐ATRP of various methacrylates using this catalyst produced good polymerization control using a 0.05 ppm loading of PC (typical loading is 1000 ppm)^[28]^ This result opens up new possibilities for the design of related PAHs exhibiting high molar absorptivities that can significantly reduce catalyst loadings. Up to this point, all the PCs described are mostly limited to the polymerization of methacrylates. In contrast to the latter, polymerization of acrylates is more challenging. Miyake et al. managed to polymerize acrylate by using rationally designed dimethyl dihydroacridines as PCs (Figure 1b).^[29]^ The process could be implemented in continuous‐flow reactors and deactivation was promoted by addition of LiBr. It is worth noticing that the outstanding performance of the PC afforded narrow‐disperse polymers with *Đ*=1.12.

In addition to all the advances developed in O‐ATRP, the use of organic photocatalysts in living polymerization reactions has also been extended to the controlled cationic polymerization of vinyl ethers. In this regard, Fors and co‐workers have explored the use of pyrylium derivatives as photocatalysts regulated by visible light (Figure 1c).[^30^, ^31^] Overall, the catalyst exhibits good performance in relatively short time‐conversions, rendering polymers with high chain‐end fidelity. Later on, to achieve higher performance both in long time‐conversion and higher oxidizing excited state, Liao and co‐workers developed a biphosphonium salt derivative, a stable PC, with tunable redox potentials. Its strong visible‐light absorption allows for the use of low catalyst loadings (down to 2.5 ppm).^[32]^

Although we have only mentioned the most representative examples here, it is impressive to note the number of advances attained in the latter years. The continuous development of PCs will lead to their eventual commercial availability and with it, further investigations and impactful advances on the monomer scope and reaction conditions. Still, some important challenges remain to be addressed: 1) higher tolerance for a wider range of functional monomers, in particular, nonconjugated monomers such as vinyl acetate to yield polymers with diverse functions; 2) ability to produce high‐MW polymers; 3) achieving polymerization using lower irradiation intensities and longer wavelength sources; 4) diminishing the catalyst loading, preferably to parts per million (ppm); 5) enhanced understanding of the photocatalyst purification (recyclability and reusability).

### 2.2 Light‐Mediated Photoacid and Photobase Generators

Photogeneration of active species such as free radicals and organic acids and bases is one of the most exciting strategies to obtain polymeric materials in a precise fashion.[^33^, ^34^] Photoinitiated reactions are performed under light irradiation, which obviates the need for elevated temperatures and solvents, thus offering an attractive alternative for industrial applications such as coatings, microelectronics, or additive manufacturing.[^35^, ^36^, ^37^, ^38^] Although photopolymerization has been mainly developed for radical polymerizations, photochemically generated organic acids and bases represent interesting alternatives for catalyzing photopolymerizations that are mechanistically based on nucleophilic substitutions, e.g., most ring‐opening polymerizations and step‐growth polymerizations.[^39^, ^40^, ^41^]

Photobase generators (PBGs) are photolabile compounds that release organic bases upon light irradiation.^[42]^ First‐generation PBGs were able to release relatively weak primary and secondary amines that served as crosslinkers,^[43]^ although these appeared unable to efficiently activate the monomers or initiators by deprotonation for the organocatalyzed anionic polymerization. Subsequent development of PBGs focused on the release of stronger bases such as amidines, guanidines, phosphazenes, and carbenes, which have been successfully used in organocatalyzed anionic polymerizations.^[44]^ The first PBG that was able to effectively generate the strongly basic 1,5,7‐triaza‐bicyclo[4.4.0]dec‐5‐ene (TBD) was reported in 2008 by Sun et al.^[45]^ The PBG was an organic salt (TBD⋅HBPh_4_) based on a protonated and thus inactive form of TBD coupled with the noncoordinating tetraphenylborate anion (BPh_4_^−^). This amidinium salt releases TBD upon 254 nm light irradiation, thus phototriggering the living polymerization of ϵ‐caprolactone (ϵ‐CL) in the presence of a 1‐hexanol initiator. The use of BPh_4_^−^‐containing PBGs has also found applications in thiol‐click polymerizations. For example, Hoyle et al. reported a thiol‐isocyanate‐ene ternary network by simultaneous activation of the thiol‐isocyanate/thiol‐ene systems. For that, a PBG composed of tributylammonium tetraphenylborate salt (TBA⋅HBPh_4_) and 2‐isopropylthioxanthone (ITX) was used. This hybrid polymerization system was able to simultaneously proceed upon 365 nm light irradiation to form highly uniform and dense thiol‐isocyanate‐ene networks.^[46]^ Most common examples using BPh_4_‐based PGBs for thiol click reactions have been described for thiol‐epoxy,^[44]^ thiol‐Michael,^[47]^ and thiol‐isocyanate,^[48]^ as well as the combination of them to form hybrid materials.[^49^, ^50^, ^51^]

On the other hand, quaternary ammonium salts composed of chromophores containing the carboxylate functionality have also been reported as versatile PBGs that are able to release a wide variety of bases via photodecarboxylation.^[52]^ The advantages of these PBGs are the commercial availability of most of the precursors, together with the easily tunable photochemical properties of the PBGs. This class of PBGs has also been exploited for thiol‐click photopolymerizations as well as ROP of cyclic esters,^[53]^ epoxides,^[54]^ and polyurethane step‐growth photopolymerization.^[55]^ In the last example, Zivic et al. reported the synthesis of novel thioxanthone‐based PBGs containing the protonated form of 1,8‐diazabicyclo[5.4.0]undec‐7‐ene (DBU) for the direct photopolymerization of polyurethane networks from alcohols and isocyanates for coating and 3D printing applications.

**Figure 2:**
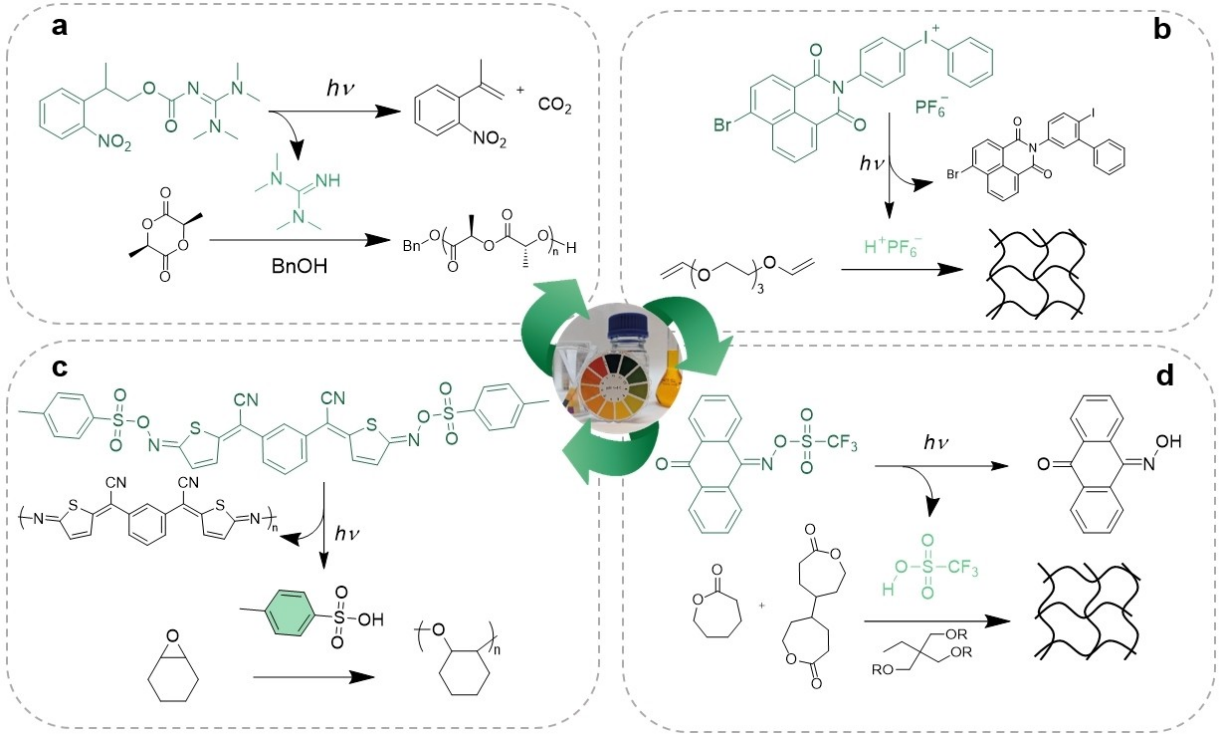
a) Scheme of the phototriggered ROP of L‐lactide using NPPOC‐TMG.^[61]^ b) Cationic polymerization of triethylene glycol divinyl ether by naphthalimide‐substituted iodonium salt.^[63]^ c) Scheme of the cationic polymerization of cyclohexene oxide using the bis‐substituted thiophene containing iminosulfonate PAG.^[64]^ d) Scheme of the polymerization of crosslinked polyester networks using an anthraquinone‐based iminosulfonate PAG.^[65]^

They successfully obtained 3D objects, although long curing times and the generation of CO_2_ from the decarboxylation upon irradiation led to defects in the final products. To avoid solubility and stability issues stemming from the ionic nature of these PBGs, non‐ionic PBGs containing labile carbamate bonds that decarboxylate upon light irradiation have also been developed.[^56^, ^57^] This type of PBG was first investigated for the photocuring of epoxy resins^[42]^ and more recently for the ROP of *N*‐carboxyanhydrides (NCAs). For the latter, Heise et al. reported a carbamate‐based PBG that releases cyclohexylamine upon 254 and 345 nm light irradiation. This primary amine was able trigger the ROP of benzyl‐L‐glutamate or *N*‐trifluoroacetyl‐L‐lysine with just 2 min of irradiation and 46 h of dark post‐treatment.^[58]^ An important step forward was achieved when Bowman et al. demonstrated that the strong 1,1,3,3‐tetramethylguanidine (TMG) base could be photocaged by a carbamate bond. The authors utilized 2‐(2‐nitrophenyl)propoxycarbonyl‐1,1,3,3‐tetramethylguanidine (NPPOC‐TMG) as the PGB in a photoinduced thiol‐Michael addition.^[59]^ The fast kinetic profile showed suitability for spatial control unachievable with less basic amines such as diethylamine.^[60]^ The development of NPPOC‐TMG was also exploited by Kuroishi and Dove for the ROP of L‐lactide (Figure 2a).^[61]^ The authors reported good dark stability, although 15 minutes of irradiation followed by 3 h in the darkness was necessary to achieve 90 % monomer conversion.^[61]^ Modifications in *o*‐nitrobenzyl (ONB) compounds were studied for visible‐light‐initiated thiol‐Michael addition.^[62]^ By incorporating an electron‐donating substituent on the ONB ring, the PBG was able to efficiently release the strong TMG base under light irradiation above 400 nm. The modified PBG was able to activate the thiol‐Michael polymerization efficiently without the generation of the undesired radicals.

In addition to PBGs, photolabile compounds that release acidic species upon light irradiation (photoacid generators or PAGs) have also been extensively investigated in polymer chemistry.[^66^, ^67^] The wide variety of acid‐responsive polymerizable monomers (epoxides, vinyl ether, oxetanes, etc.) enabled PAGs to be used in a myriad of applications such as coatings, adhesives, inks, and semiconductors, among others.^[68]^ The most industrially employed PAGs are the onium salts discovered in the 1970s by Crivello and co‐workers.[^69^, ^70^] These are able to efficiently release H^+^ under high‐energy‐light irradiation (<300 nm), which limits their use to several applications.^[71]^ The strategy of red‐shifting the absorption spectra of iodonium salts has been pursued, for instance, by the use of a naphthalimide chromophore, which enabled the polymerization of bis‐epoxide and divinyl ether with 365 and 385 nm light irradiation with high monomer conversion using short irradiation times (<2 min) (Figure 2b).^[63]^ In another study, Dove et al. used a triarylsulfonium hexafluorophosphate salt for the ROP of the cyclic esters ϵ‐CL, δ‐valerolactone (δ‐VL), and trimethylenecarbonate (TMC) at 365 nm, although low polymerization rate and loss of control was observed above 50 % monomer conversion.^[72]^ Non‐ionic PAGs have also been reported to improve stability and solubility in organic solvents and monomer mixtures. In this regard, imino and imidosulfonates have been reported.[^66^, ^73^, ^74^, ^75^] These PAGs release sulfonic acids upon light‐induced homolytic dissociation of their N−O bonds followed by proton abstraction from the surrounding media.[^74^, ^76^] Recently, a bis‐substituted thiophene‐based chromophore containing iminosulfonate was described as a non‐ionic PAG capable of polymerizing epoxides and vinyl esters upon 365–475 nm light irradiation. Complete conversion was achieved within 1 min using 1 wt % of PAG for the polymerization of an epoxide formulation (Figure 2c).^[64]^ More recently, Sardon et al. reported the use of six novel PAGs based on imino and aryl sulfonates, bearing anthrone or anthaquinone chromophores, which were to release triflic, *para*‐toluenesulfonic, and methanesulfonic acids. The reported catalysts were able to trigger the acid‐catalyzed ROP of ϵ‐CL either in solution or in bulk with fast polymerization rates achieved at 100 °C. Moreover, polyester‐based crosslinked coatings were obtained using the reported catalyst, interestingly extending the number of coatings achievable by photopolymerization (Figure 2d).^[65]^

In all, PAGs and PBGs offer a potentially interesting approach to create complex materials due to the external spatiotemporal control afforded by the photogeneration of active species, which enables polymerizations to be carried out remotely. Liberation of strong organic bases and acids allows for the polymerization of distinct monomers to afford polymers bearing features difficult to achieve by conventional radical photopolymerization such as biodegradability and recyclability. Nonetheless, further advances in terms of developing PBGs and PAGs with 1) superior base/acid release profiles, 2) efficient liberation of species under visible light, and 3) wider accessibility and modest costs to promote their practical use, are sought after.

### 2.3 Photoswitchable Organocatalysts

**Figure 3:**
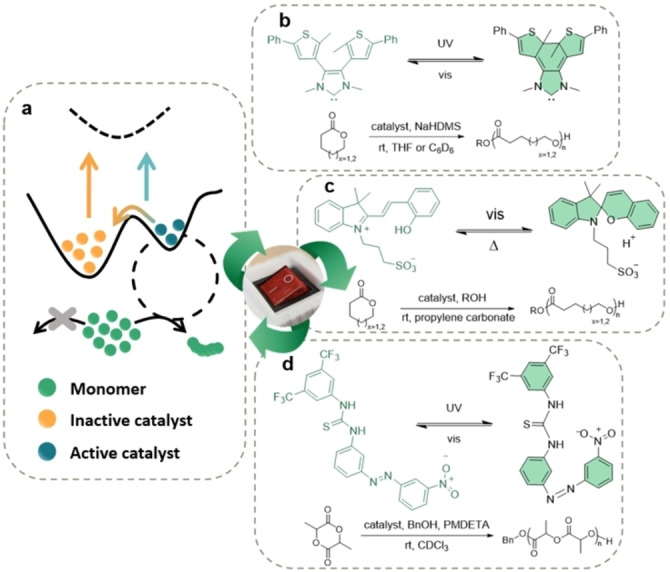
a) Representation of photoswitchable catalysis. b) Photoinduced π‐electrocyclization of a diarylethene.^[84]^ c) Reversible reaction of a spiropyran‐based photoswitch.^[85]^ d) Light induced *cis*–*trans* isomerization of an azobenzene‐based thiourea.^[86]^

On‐demand design and synthesis of polymers with controlled composition, structure, and length is a key factor in the creation of new materials with tailored properties.^[77]^ In this regard, the ad hoc preparation of advanced polymer materials is directly related to the development of more versatile catalytic systems. Catalysts capable of externally modulating their activity towards polymer synthesis have gained a lot of attention in the last years, especially in the area of light‐driven catalysis. Although other external stimuli such as temperature^[78]^ or redox potentials[^79^, ^80^] have been leveraged to modulate catalytic activity, the use of light is particularly interesting due to its improved spatiotemporal control, precise wavelength tunability, and operational ease.^[81]^ In this sense, photoswitchable catalysts are formed by combining a catalytically active component bonded to a photochromic unit. The challenge in developing photoswitchable organocatalysts lies in the design and preparation of photoactive molecules that provide efficient photochemical performance and readily tunable activity/selectivity in both states. In this regard, photoswitchable catalysts that are able to amplify the catalytic activity difference between the two states (ON/OFF) are the favored approach (Figure 3a).[^82^, ^83^] It has to be noted that photoswitchable catalysts could in principle modulate their control towards regio‐, stereo‐, and chemoselective reactions, although these selectivities are challenging to achieve and scarcely investigated. Although reversible catalytic efficiency of the photoswitches covered in this section rely on the p*K*_a_ difference between states and thus could have been addressed in the previous section, photoswitchable organocatalysis goes one step beyond, as it offers the potential to regulate the catalytic efficiency on demand. Here, the most relevant examples of photoswitchable organocatalysts employed for polymerization will be discussed.

Photoswitchable catalysis for ROP was first reported by Osaki and co‐workers in 2009, whereby they exploited the *cis*–*trans* isomerization of the cinnamoyl group in a modified α‐cyclodextrin.^[87]^ The UV‐induced conformation change blocked the active site of the catalyst, substantially lowering its activity towards δ‐VL ROP. After UV‐induced inactivation of the catalyst, only 12 % monomer conversion was observed after 24 h at 100 °C, whilst 82 % conversion was achieved with the active isomer under the same conditions. However, the long irradiation times needed to lower the catalytic activity remains a major drawback. More recently, in 2013, Bielawski et al. reported the use of a photoswitchable organocatalyst for the ROP of cyclic lactones (Figure 3b).^[84]^ The polymerization was catalyzed by an *N*‐heterocyclic carbene (NHC) that was integrated into the core of a diarylethene moiety. Upon UV light irradiation, the catalyst switched from the open active isomer to an inactive closed form that lowered the electron density in the carbene, thereby reducing its nucleophilicity. Significant attenuation of the polymerization activity under UV irradiation was observed for δ‐VL (*k*_amb_/*k*_UV_=59). Nevertheless, the slow and incomplete photoswitching reaction from the active to inactive state is not ideal, as it limits temporal control over the polymerization reaction, especially when kinetically rapid reactions are involved. Furthermore, the formation of insoluble byproducts proved deleterious to the photoswitchable cycles and reduced the activity of the catalyst after each iteration. In this regard, subsequent efforts have focused on creating a photoswitchable guanidine catalyst for the ROP of L‐lactide, albeit low catalytic efficiency was observed due to the low basicity of the active form.^[88]^ In a more recent study, Boyer and co‐workers reported a novel reversible spiropyran‐based photoswitch for the cationic ROP of cyclic esters (Figure 3c).^[85]^ Irradiation with blue LED light (*λ*=460 nm) induces the formation of the spiropyran, whereupon a proton is be released to catalyze the ROP of ϵ‐CL and δ‐VL. Once the light source is removed, the system reverts to the inactive form, albeit via a very slow process, i.e., 8 h to convert 65 % of the catalyst to its inactive form. The catalyst is also poorly soluble in organic solvents, which limits the scope of its application. In an effort to overcome such reversibility and solubility issues, Read de Alaniz and co‐workers developed a modified spiropyran‐based photoswitch containing *t*‐Bu groups on the phenolic moiety.^[89]^ By making these structural modifications, the authors claimed much faster relaxation kinetics that led to enhanced polymerization control. Nevertheless, long reaction times and high catalytic loadings were needed to obtain high monomer conversion.

Classic azobenzene‐based photochromism has been also exploited for the organocatalyzed ROP of L‐lactide by Wu and co‐workers (Figure 3d).^[86]^ The photoinduced *E*–*Z* isomerization of the azobenzene fragment under UV light (365 nm) positions the nitro group proximal to the thiourea moiety, forming H‐bonds and thereby blocking the active site of the catalyst. Catalyst activity can be regenerated by irradiation with visible light. The main disadvantage is that the active form corresponds to the more stable isomer and thus the catalyst has to be irradiated continuously to retain the inactive state, a process that is too energy intensive. Furthermore, inactivation is only partially achieved under UV irradiation (i.e., 34 % in the inactive form), and this is compounded by the fact that the photoswitching rate is comparable to the polymerization kinetics. Taken together, the degree of light‐induced control afforded by this reaction is less than optimal.

Undoubtedly, the advent of photoswitchable organocatalysts has opened up opportunities for enhanced control over polymerization reactions and significant advances can be expected in the coming years. The examples described herein represent the tip of the iceberg in terms of potential possibilities, although it is clear that improvements are still needed in the implementation of photoswitches that show 1) rapid switching between states with negligible side reactions to enhance the spatiotemporal control conferred by the catalysts, 2) sufficient activity differences between states, which might be challenging to achieve in a single molecule, and 3) extended scope of polymerizable monomers to include radical or cationic polymerizations, both of which remain unexplored so far.

### 2.4 Stereocontrolled Polymerization

Stereocontrolled polymerization techniques have provided excellent control over polymer stereochemistry without sacrificing the control in the polymer length and chain end functionality.^[10]^ Besides, the stereoregularity of a polymer is responsible for its crystallinity, and consequently, for the fact that its physical and thermomechanical properties are superior to those of non‐stereoregular polymers. This is because differences in tacticity lead to profound differences mainly in the melting (*T*_m_) temperature.^[90]^ Recent advances in coordination–addition, anionic, cationic, and radical polymerizations have enabled access to polymers with isotactic, syndiotactic, and heterotactic structures.^[91]^ Indeed, several polymers can now be prepared with relatively high degrees of stereocontrol including certain (meth)acrylates, (meth)acrylamides, vinyl ethers, and vinyl oxazolidinones.

Given their excellent ability in transferring chirality to the polymer backbone, and the countless ligand/metal combinations, transition‐metal‐based catalysts have dominated the field of stereocontrolled polymerization since 1950. For instance, since the discovery of commodity polyolefins, several advances toward the stereocontrolled copolymerization of propylene with functional monomers have been made using metal catalysts. The coordination–insertion polymerization of α‐olefins, first accomplished by Ziegler and Natta,[^92^, ^93^] has resulted in a number of transition‐metal‐based catalytic systems that offer exquisite levels of stereocontrol. Nevertheless, the metal catalysts utilized are often incompatible with the incorporation of polar functional groups into polyolefins, and the stereoinduction is almost exclusively limited to nonpolar monomers.[^94^, ^95^] To combat this challenge, late‐transition‐metal catalyst have been used although stereocontrolled examples of copolymerization of α‐olefins are rare,[^96^, ^97^] and to the best of our knowledge no examples of organocatalyzed stereocontrolled vinyl polymerizations have been reported in the literature.^[91]^

Regarding cationic vinyl polymerizations, Leibfarth and co‐workers showed that a specially designed 1,1′‐bi‐2‐naphthol (BINOL)‐based phosphoric acid ligand could be used to control the stereochemistry and microstructure of different poly(vinyl ethers) in combination with TiCl_4_. Nevertheless, no polymerization was achieved when only the chiral phosphoric acid was used as catalyst.^[98]^ The resulting polymers are not only semicrystalline but also mechanically strong and highly adhesive.

**Figure 4:**
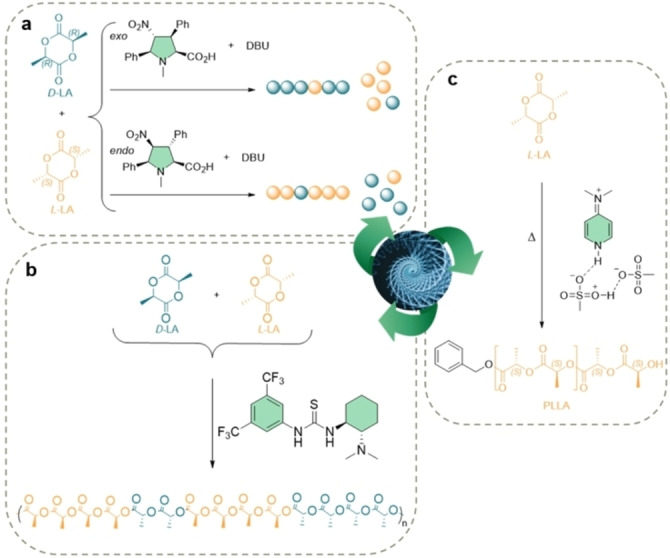
a) Enantioselective ROP of *rac*‐LA by catalyzed by a highly substituted amino acid.^[99]^ b) Stereoselective organocatalyzed ROP of *rac*‐LA by an amino‐thiourea organocatalyst.^[100]^ c) Stereoretentive ROP of L‐lactide, as catalyzed by DMAP:MSA.^[101]^

Another class of polymers in which stereocontrol is of great interest are polyesters, especially poly(lactide) (PLA). Metal alkoxides and some achiral organometallic catalysts have been used to mediate the stereocontrolled polymerization of racemic lactide (*rac*‐LA) and subsequent formation of stereoregular PLA.^[102]^ Nevertheless, increasing environmental concerns, together with rising interest in metal‐free polymer products, have driven the development of organocatalysts that can perform stereocontrolled polymerization.[^2^, ^103^] As with metal‐based catalysis, stereoselective ROP of *rac*‐LA to afford isotactic‐enriched semicrystalline PLA can be mediated by organic achiral species bearing sterically bulky moieties, such as NHC,^[104]^ and dimeric^[105]^ or cyclic trimeric^[106]^ phosphazene bases. On the other hand, chiral organocatalysts including binaphthol‐type phosphoric acids,^[107]^ or a β‐isocupreidine/thiourea/chiral binaphthylamine,^[108]^ have also been investigated. In a more recent report, Columbier et al., reported the ability of achiral TBD to produce greatly isotactic PLA from *rac*‐LA at −75 °C.^[109]^ Both chiral and achiral catalysts showed excellent stereocontrol; however, the ROP reactions are generally conducted at low temperatures which limits their use. Recently, the use of organocatalysts based on hydrogen‐bond donor/acceptor adducts have been proposed to address the stereoselective polymerization at more elevated temperatures. Thus, bifunctional systems based on amino‐prolines or amino‐thioureas have been proposed.[^99^, ^100^] In the former example, two diastereomeric densely substituted amino acids were employed for the room‐temperature stereocontrolled ROP of *rac*‐La in combination with DBU (Figure 4a). In an approach based on amino‐thiourea, Taton et al., explored the use of both enantiomeric chiral thiourea‐amine Takemoto's catalysts to trigger the isoselective polymerization of racemic lactide at room temperature (Figure 4b).^[100]^ Also, mono‐ or bis‐ureas in combination with organic bases could operate at high temperatures while maintaining good control over the polymerization. Several other hydrogen‐bond acceptors, including amido‐indoles and fluorinated alcohols,[^110^, ^111^] have also been reported to be acceptable replacements.

In an effort to avoid the undesired epimerization and transesterification reactions usually associated with the high‐temperature ROP of LA, which reduce the thermal and mechanical properties of the resulting polymer, Sardon et al., explored the use of a nonstoichiometric acid–base mixture catalyst prepared by mixing methanesulfonic acid (MSA) and 4‐(*N*,*N*‐dimethylamino)pyridine (DMAP).^[101]^ This catalyst allowed the bulk preparation of stereoretained PLLA through ROP and at elevated temperatures up to 180 °C (Figure 4c). The robust thermal stability of the organic acid–base mixture limits the incidence of side reactions, e.g., epimerization, transesterification, and macrocyclization. This, together with its low volatility, makes this catalyst system suitable for industrial applications.

Despite these significant advances, this area of polymer chemistry has lagged behind in terms of the ability to control MWs, *Đ*, and composition. Thus, only a few methods have been developed to the level of commercial viability. It is worth noticing that only a few organocatalysts have shown the ability to promote stereocontrolled polymerizations and the monomer scope is even more limited, and much of the underlying stereocontrol mechanisms remain unknown. Exciting opportunities still exist to address outstanding challenges in designing active organocatalysts that exhibit 1) both high stereoselectivity and catalytic activity, 2) control over polymer tacticity, 3) high thermal stability that can withstand industrially relevant melt‐processing conditions, and 4) the ability to polymerize an even wider range of monomers to increase the diversity of novel functional materials.

### 2.5 High‐Temperature Polymerizations/ Depolymerizations

(De)polymerization processes of chemically stable and poorly soluble polymeric materials have generally required thermally robust metal salt catalysts (zinc or lead acetates, sodium/potassium sulfate, or titanium phosphate) and very harsh conditions such as microwave assistance or supercritical water conditions.^[3]^ However, metal‐based catalysts are challenging to separate from the crude product and have poor selectivity, which makes industrial scale‐up difficult to achieve. As an emerging alternative, organic bases (i.e., TBD, DBU, or DMAP) have proven themselves to be effective. One of their drawbacks is that they show poor thermal stability at temperatures essential for both polymer recycling and formation,[^4^, ^5^] whereupon they are fully or partially degraded, resulting in diminished performance and undesirable side reactions.^[112]^ A solution to this problem is to employ thermally stable ionic liquids, Brønsted acid ionic liquids, or amidinium or guanidinium salts, including the benzoate and mesylate salts of DBU and TBD, respectively, which are trivially prepared via the acid–base reaction between an organic acid and the organic base. Moreover, both stochiometric and nonstoichiometric acid–base mixtures can be easily prepared. These mixtures offer a wide range of possibilities, since their dual functionality (i.e., amphoteric nature) can be fine‐tuned by adjusting the stoichiometry and chemical nature of the compounds.^[113]^

**Figure 5:**
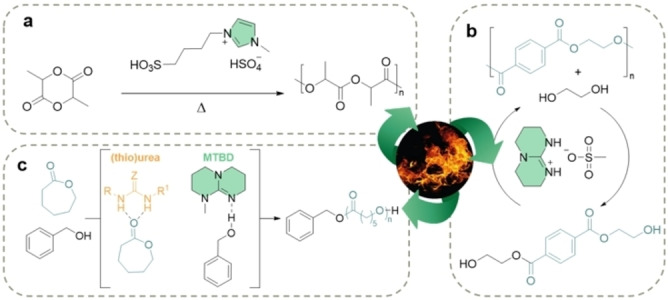
a) Ring‐opening polymerization of L‐lactide using BAIL.^[117]^ b) The depolymerization of PET and repolymerization of monomeric products at high temperatures.^[115]^ c) H‐bonding‐ and imidate‐mediated ROP of ϵ‐caprolactone (CL).^[122]^

Furthermore, they have been successfully used in industrially relevant conditions that require high temperatures, such as the preparation of polyesters, polyesteramides, or polyethers.[^114^, ^115^] For example, Brønsted acid ionic liquids (BAILs) such as 1‐(4‐sulfobutyl)‐3‐methylimidazolium hydrogen sulfate have been utilized to promote polyesterification^[116]^ and ROPs^[117]^ (Figure 5a), and urea‐based ILs^[115]^ have been used for depolymerization of polyamides and polyesters.^[3]^ Similarly, acid–base mixtures of DBU and benzoic acid (BA) have been employed for ROP polymerization of lactide.^[118]^ TBD and methanesulfonic acid (MSA) mixtures^[115]^ have demonstrated both thermal stability and efficacy for the depolymerization of poly(ethylene terephthalate) (PET) or bisphenol‐A polycarbonate (BPA‐PC).[^119^, ^120^] These organic salts were also found to efficiently promote the polymerization of ethylene glycol and dimethyl terephthalate, while producing PET (Figure 5b) at high temperatures with high molecular weights and higher crystallinity than provided by a titanium(IV) butoxide catalyst. Similarly, Peruch et al. prepared some DMPA‐MSA organic salts to promote the ROP of L‐LA at elevated temperatures.^[121]^ On the other hand, multi(thio)urea H‐bond donors with amino cocatalysts (e.g., 7‐methylTBD or mTBD) exhibit fast kinetics, excellent control, and good thermal stability with insensitivity to the reaction conditions (Figure 5c).^[122]^

On the basis of these examples, a number of innovations in the academic world are required to successfully translate new catalytic strategies to industry. In our view, the most important remaining challenges that need to be overcome in the area of (de)polymerization reactions would be: 1) Reactivity rates and MWs achieved in organocatalyzed polymerizations are low compared to metal‐based catalysts; 2) The majority of the most efficient catalysts are based on expensive compounds like TBD, which makes their implementation in industry economically difficult; 3) In contrast to metal‐based catalysts, organocatalyst loadings are often much higher, thus limiting its applicability in industry.

Therefore, to achieve optimal catalysis and to design more efficient organocatalytic systems, synthetic advances as well as a deeper mechanistic understanding of how the acid–base mixtures work, will be needed.

### 2.6 Transition‐Metal‐Free Synthesis of π‐Conjugated Polymers

In contrast to commodity polymers that feature aliphatic backbones, available methods for the transition‐metal‐free synthesis of π‐conjugated polymers (CPs) remain extremely scarce. Conceptually, this is a natural consequence of differences in the mechanisms by which the two distinct classes of polymers are formed. Since aliphatic polymers such as polyolefins are usually prepared via the radical polymerization of olefin monomers, extending the synthetic methodology to photoredox and photoinitiated polymerizations does not involve major conceptual deviations from radical‐based chemistry. CPs, however, are predominantly synthesized by transition‐metal‐catalyzed cross‐coupling, which is conceptually dissimilar since this mechanism does not involve any radical intermediates. Consequently, transition‐metal‐free and photochemical methods for the synthesis of π‐conjugated polymers remain a relatively new and emerging research area that is still rich in possibilities. In recent years, there have been independent efforts by the groups of Kalow and Yagci that can be expected to pave the way towards further advances.[^123^, ^124^, ^125^, ^126^, ^127^]

**Figure 6:**
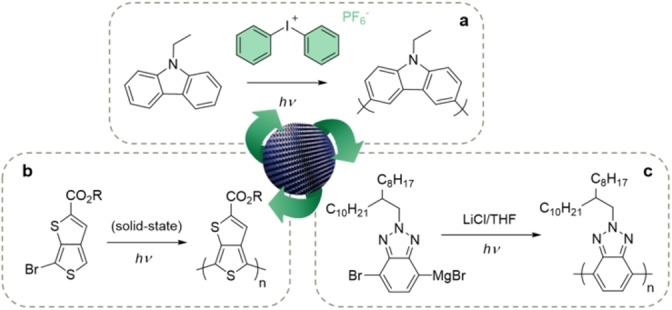
a) Oxidative radical photopolymerization.^[127]^ b) Reductive photopolymerization.^[129]^ c) Cationic oxidative photopolymerization.^[123]^

Existing transition‐metal‐free photopolymerization methods (Figure 6) for the preparation of CPs can be classified into three main categories: 1) oxidative radical photopolymerization, 2) cationic oxidative photopolymerization, and 3) reductive photopolymerization.^[124]^ The first class of photopolymerizations is primarily applicable to electron‐rich monomers such as thiophenes and carbazoles, which are used to synthesize *p*‐type (i.e., hole‐transporting) CPs. Here, the mechanism involves an initial photoinduced single‐electron transfer (SET) from the monomer to an oxidant, followed by radical combination to form new C−C bonds between the monomeric units. Representative syntheses by Yagci et al. have featured photopolymerization of *N*‐ethylcarbazole, with the aid of a diphenyliodonium salt in the seminal example,^[128]^ and subsequently in a single‐component capacity as part of a methodological improvement (Figure 6a).^[127]^ For the second class of photopolymerizations involving a cationic oxidative mechanism, Yu and co‐workers recently described the metal‐free, light‐promoted solid‐state polymerization of bromothienothiophene monomers to afford ultralow‐bandgap PTT (Figure 6b) with higher charge carrier mobilities than control polymers made via Pd‐catalyzed polycondensation.^[129]^ The superior electronic properties are thought to result from the absence of transition‐metal residues, which are frequently left behind following metal‐catalyzed cross‐coupling.

Finally, in contrast to the above transition‐metal‐free photopolymerization methods that primarily afford *p*‐type organic semiconductors, reductive photopolymerization was recently shown to be a viable strategy for obtaining *n*‐type (i.e., electron‐conducting) polymers. Kalow et al. demonstrated that electron‐deficient Grignard monomers could be polymerized in the presence of LiCl when irradiated with visible light (Figure 6c).^[123]^ Aside from the chain‐growth and transition‐metal‐free character of the polymerization, another intriguing aspect was that the resulting polymer molecular weights were dependent on the irradiation wavelengths used. In‐depth experimental and computational studies have revealed that the photopolymerization involves Mg‐templated radical‐nucleophilic aromatic substitutions (S_RN_1), whereby the incoming Grignard monomers are coordinated to a growing radical anion chain before coupling with it.^[123]^

All in all, the transition‐metal‐free synthesis of CPs is still in its infancy, and many exciting discoveries can be expected to surface in the coming years. In particular, the ability to tailor the molecular structures of custom‐made monomers will allow for the exploration of CPs of increasing structural diversity and properties made using this method. Furthermore, as our mechanistic understanding of these transition‐metal‐free photopolymerizations increases, it may soon become possible to exercise greater control over the polymerization conditions as well as the end results. At the moment, most of the contemporary noble‐metal‐free methods for CP synthesis center on the polymerization of electron‐rich monomers to give *p*‐type CPs. Extension of these methodologies to *n*‐type and donor–acceptor‐type CPs can thus be anticipated, and the Kalow group's recent efforts[^128^, ^129^] on the former represent an ideal launchpad for further developments.

Increased diversity in CP architectures can also be expected since the development of chain‐growth photopolymerizations will enable access to π‐conjugated block copolymers and surface‐grafted CPs that cannot be provided by conventional Pd‐catalyzed polycondensation methods. Finally, with all the rapid ongoing developments in organic photoredox catalysis, there may yet be opportunities to investigate the use of organic photoredox catalysts for transition‐metal‐free, visible‐light‐driven CP synthesis. Aside from the inherent appeal of sustainable chemistry, the ability to synthesize CPs without transition metals also offers the added advantage of providing polymer products that are free of metallic residues that could negatively impact their optoelectronic properties.

### 2.7 Sequence‐Controlled Polymers

Sequence‐controlled polymers (SCPs) are a class of copolymers whereby chemically distinct monomers are specifically organized or arranged within the polymer chain.^[130]^ Nature has demonstrated that sequence control in biopolymers is an essential feature implicated in heredity, self‐replication, self‐assembly, and molecular recognition. Although biochemistry and biophysics have advanced significantly in the artificial synthesis of sequence‐defined biopolymers by protein engineering[^131^, ^132^, ^133^] and by DNA‐templated polymerization,[^134^, ^135^, ^136^] the non‐natural manufacture of SCPs is still in its infancy. Chemical approaches for the synthesis of SCPs emerged during the 2010s,[^130^, ^137^] and traditionally have been classified according to the polymerization approach.[^138^, ^139^] These categories involve: 1) step‐growth polymerizations of bifunctional monomers,[^140^, ^141^, ^142^] 2) chain‐growth mechanisms using living radical polymerization,[^143^, ^144^, ^145^] and 3) multistep‐growth polymerization following stepwise chemical methods.[^146^, ^147^, ^148^] However, most of these methods offer low control over the polymer architecture or require tedious steps to afford the desired materials.

**Figure 7:**
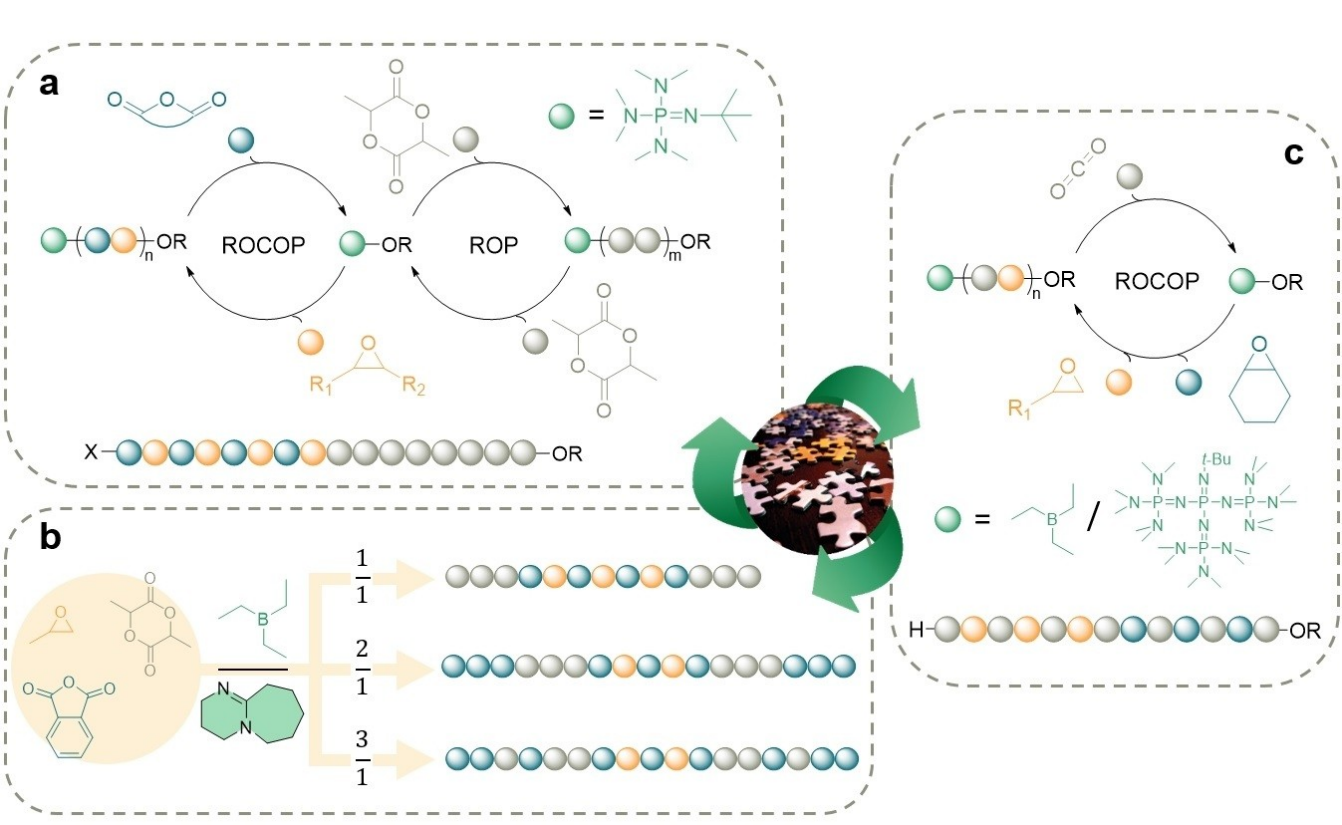
a) Self‐switchable copolymerization of epoxides and anhydrides with lactide under phosphazene base catalysis.^[158]^ b) Representative example of tandem organocatalyzed terpolymerization of mixtures of PO, PA, and *rac*‐LA.^[160]^ c) Anionic copolymerization of epoxides and CO_2_ with Et_3_B/^t^BuP_4_ as a dual catalyst.^[161]^

To fulfill sustainability demands, ester or carbonate‐based copolymers exhibit chemistries that lend themselves to facile depolymerization and recycling, allowing monomer recovery for new feedstocks.[^149^, ^150^] Since the early 2000s, ROP of lactones and other strained cyclic monomers provides an efficient route to thermoplastics derived from renewable resources.[^151^, ^152^] In the past decade, switchable polymerization catalysis has become a powerful tool to SCPs from monomer mixtures.[^153^, ^154^] In these polymerizations, the catalyst “switches” from ring‐opening copolymerization (ROCOP) of heterocycles/heteroallenes to ROP of heterocycles, leading to sequence‐controlled block copolymers. Recently, Williams has reviewed the scope of switch catalysis emphasizing the potential applications of the materials obtained.^[154]^ Switch polymerization has been successfully achieved by using metal‐based catalysts such as dinuclear macrocycles,^[155]^ salen complexes,^[156]^ or Zn^II^‐β‐diiminates.^[157]^ However, the resulting materials are not suitable for biomedical applications, since the complete removal of trace metal contaminants is often impossible. In 2018, an alternative metal‐free approach was reported. The use of a phosphazene base (^t^BuP_1_) provided sequence control in the copolymerization of epoxides and anhydrides with lactide (Figure 7a).[^158^, ^159^]

This self‐switchable metal‐free route is versatile and leads to diblock, triblock, pentablock, or star‐block polymers depending on the alcohol used as initiator. Later on, ^t^BuP_1_ combined with triethyl borane (Et_3_B) was also used as a two‐component catalyst, offering high tunability in the preparation of polyester‐polyether SCPs.[^162^, ^163^] Wang and co‐workers explored the performance of Et_3_B combined with DBU for switch polymerization.^[160]^ The resulting catalyst pair is highly selective for terpolymerization of mixtures of PO, PA, and *rac*‐LA, proving that organocatalysis renders materials with high degrees of structural complexity from monomer mixtures (Figure 7b). They also proved the versatility of this organocatalyzed switch polymerization in combination with concurrent RAFT polymerization, obtaining triblock quaterpolymers from monomer mixtures of epoxides, anhydrides, *ra*c‐LA, and vinyl monomers.^[164]^ In 2016 Gnanou and Feng successfully prepared alternating polycarbonates by a metal‐free approach using Et_3_B/^t^BuP_4_ tandem.^[165]^ Later on, they expanded the scope of this system, including the copolymerization of epoxides with CO_2_ (Figure 7c).^[161]^ The obtained well‐defined triblock copolymers exhibited different rubber properties, depending on the poly(cyclohexene carbonate) content.

In addition to chemical recyclability and reprocessing, switch polymerization offers an excellent route to SCPs derived from monomer mixtures, achieving high control over the primary structure. Organocatalysts exhibit good performances when compared to metal‐based catalysts, but the future challenges would be focusing on: 1) improving the monomer tolerance and scope, 2) improving the molar mass values of the materials obtained, and 3) reducing the catalyst loading.

### 2.8 Accelerating the On‐Demand Dynamic Covalent Bond Exchange

Traditionally, thermoplastics and thermosets have covered the polymeric scope for plastic materials. Whilst thermoplastics are constituted by entangled, linear polymer chains, thermosets are defined by covalently bonded networks. Such a crosslinked structure results in robust, inert, and insoluble materials, which makes them ideal candidates for high‐temperature or structural applications.^[166]^ However, given the irreversible nature of the chemical crosslinks, their reprocessability is limited and their recyclability difficult to achieve. Starting with the pioneering work by Bowman^[167]^ and Leibler^[168]^ in the mid‐2000s, the introduction of dynamic covalent chemistry (DCC)^[169]^ to polymer science has opened the field to covalent adaptable networks (CANs) and vitrimers.[^170^, ^171^, ^172^] The dynamic nature of their crosslinks leads to “smart” materials as they exhibit the robustness of thermoplastics but yet, they are also reprocessable, recyclable, and reconfigurable, upon external stimuli such as temperature changes,[^173^, ^174^, ^175^] light radiation,[^167^, ^176^, ^177^, ^178^, ^179^] or molecular triggers.^[180]^ The scope of dynamic crosslinks utilized for CANs fabrication is wide and varies from addition–fragmentation chain‐transfer reactions to transesterifications and Diels–Alder processes, among others.^[181]^

**Figure 8:**
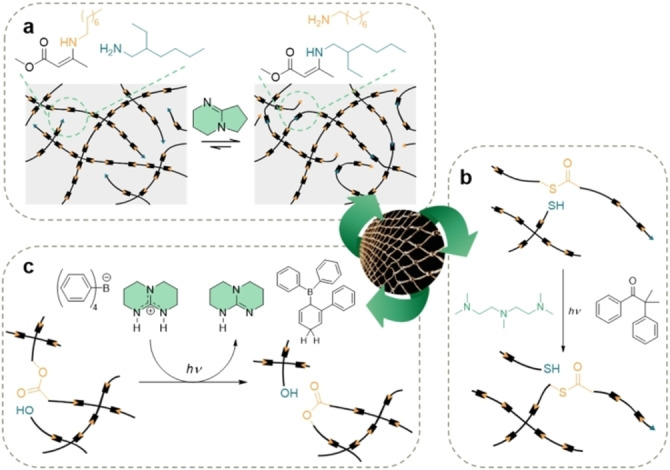
a) Organomediated dynamic vinylogous urethane amine exchange with model reaction (inset).^[185]^ b) Bistable light‐mediated phase switching of thiol‐thioester exchange in the presence of a base.^[186]^ c) Photobase‐triggered network rearrangement process of a vitrimer based on dynamic transesterification.^[187]^

In this regard, organocatalysts have proven to effectively trigger the dynamic properties of CANs and vitrimers when used as additives within the polymeric matrix (defined as external catalysis).^[180]^ Brønsted or Lewis acids or bases can trigger the exchange process upon heating at threshold temperatures,[^182^, ^183^, ^184^] turning the polymeric material into a fluid state for processing. An interesting result published by Du Prez and co‐workers shows the precise control of the amine exchange of vinylogous urethane vitrimers by using acids (*p*‐toluenesulfonic acid (p‐TSA) or sulfuric acid) or bases (1,5,7‐triazabicyclo[4.4.0]dec‐5‐ene (TBD) or 1,5‐diazabicyclo[4.3.0]non‐5‐ene (DBN)) leading to programmable materials with predictable viscoelastic properties (Figure 8a).^[185]^

Nonetheless, organocatalysis has also played a key role on the development of the first bistable switching material. Conventionally, stimuli‐responsive materials quickly return to the solid state once the stimulus is removed. In 2018, Bowman and co‐workers presented a responsive thiol‐thioester exchange‐based polymer network containing a UV‐photoinitiator (DMPA) and a weakly basic amine (*N*,*N*,*N*′,*N*′′,*N*′′‐pentamethyldiethylenetriamine, PMDTA).^[186]^ Hence, at room temperature and in the absence of a base, the thiol‐thioester exchange requires a high activation enthalpy and the reaction does not take place (Figure 8b). In addition, the versatility of organic chemistry provides access to the development of stimuli‐responsive organocatalysts. Schlögl and co‐workers reported a photolatent base for the local control of dynamic exchange reactions in crosslinked thiol‐epoxy networks (Figure 8c).^[187]^ Upon light exposure, the quaternary ammonium salt undergoes a cascade disassembly which results in the release of TBD.^[188]^ The specific photoactivation of the organocatalyst permits the transesterification dynamic exchange only in the light‐exposed areas while keeping intact the covered areas.

Nonetheless, organic chemistry and organocatalysis has gone one step further. Recently, Du Prez has reviewed how internal catalysis may be a powerful tool for the design of a new generation of CANs and vitrimers.^[189]^ By smart monomer design, one can introduce neighboring catalytic groups that enhance the reactivity of the dynamic covalent bonds by stabilizing the transition states, thus lowering the kinetic barriers for the rearrangement process (neighbor group participation or NGP). In contrast to external catalysis, this rational approach aims to mimic the sophisticated enzymatic processes in nature.^[190]^ The most common approach for catalyzed NGP on CANs relies on the presence of nucleophilic or basic vicinal atoms, such as sulfur,^[191]^ oxygen,[^192^, ^193^, ^194^, ^195^, ^196^, ^197^, ^198^, ^199^] and nitrogen.[^200^, ^201^, ^202^, ^203^, ^204^]

The discovery of CANs and vitrimers opened the field for self‐healable and smart materials, addressing the need for reprocessing and recycling of “plastics”. Organocatalyzed reforming not only offers good performances but also allows the preparation of bio‐related materials, lowering the potential toxicities whilst keeping the structural properties. The introduction of NGP in the monomer design is expected to increase the efficiency of CANs reprocessing, broadening the scope of polymeric materials and future applications.

### 2.9 Polymer Upcycling via Organocatalyzed and/or Transition‐Metal‐Free C−H Functionalization

Aside from recycling commodity polymers and designing novel recyclable polymers, polymer upcycling[^205^, ^206^] is another attractive way to inject value into postconsumer plastic waste that would otherwise be destined for a landfill or an incinerator. By chemically modifying the molecular structure of commodity polymers, it becomes possible to leverage their low‐cost, high‐volume industrial production to create novel value‐added materials for a host of applications that are unrelated to the original one. Strategies for upcycling polymers typically involve postpolymerization modification, i.e., chemical functionalization of the polymer backbone with moieties that confer new physicochemical properties not found in the precursor polymer. While postpolymerization functionalization of polymers is certainly not new,^[122]^ the field nonetheless remains relatively underdeveloped. Given the type of molecular structures presented by most commodity polymers (e.g., PE, PP, PS), postpolymerization modification essentially involves functionalization of C−H moieties along the fully saturated aliphatic backbone or pendant aromatic rings (if present), i.e., replacing H with different functional groups that confer desired properties.^[207]^

**Figure 9:**
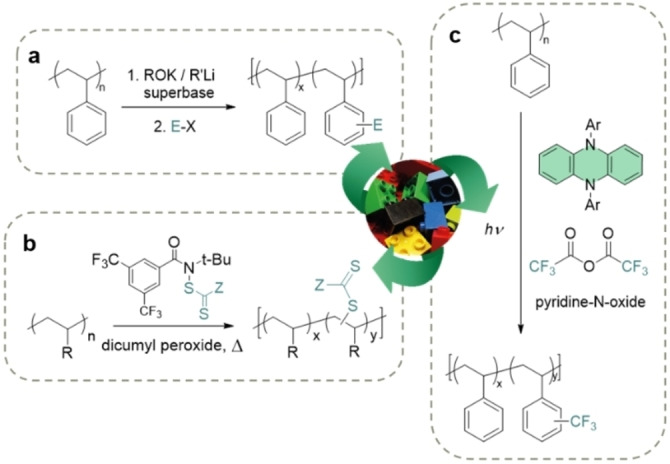
a) Representative past effort on the C−H functionalization of polystyrene by using a superbase.^[211]^ b) Metal‐free xanthylation of branched polyolefins.^[219]^ c) Organocatalyzed trifluoromethylation of PS.^[220]^

Early C−H functionalization efforts often entailed subjecting the polymers to crude and unselective reactions[^208^, ^209^] that have limited utility in the modern context, especially in regard to polymer upcycling. Even though the field of organic synthesis has progressed significantly over the years, many of the new synthetic methodologies remain untested on polymers. In the context of post‐functionalizing aromatic commodity polymers, there have been sporadic efforts in past decades by some research groups, notably those led by Sawada^[210]^ and Fréchet^[211]^ in the 1990s, and more recently, by Leibfarth and co‐workers.^[212]^ Prior work by Fréchet et al. enabled the stepwise introduction of various functionality,^[211]^ but those methods necessitated the use of superstoichiometric amounts of *n*‐BuLi/*t*‐BuOK, superbase. (Figure 9a). Subsequent contributions by other groups (Vlcek, Register, Jannasch)[^213^, ^214^, ^215^] focused on facilitating the introduction of hydrophilic moieties onto PS, but the methodologies similarly required *n*‐BuLi, a reagent whose relative high cost and pyrophoric nature would make it prohibitive to use on larger scale.

In recent years, modern C−H functionalization methodologies have also been increasingly utilized for modifying commodity polymers.[^216^, ^217^, ^218^] However, one of the main drawbacks of these methods is the need for metal‐based catalysts based on nonrenewable and/or toxic noble metals such as ruthenium. Reliance on such catalysts would detract greatly from the overall “green” theme of upcycling plastic waste. A potential solution is thus to leverage metal‐free C−H functionalization strategies that rely on organocatalysts and/or all‐organic reagents. On that front, the efforts of Leibfarth and co‐workers have resulted in several interesting examples. In 2019, Leibfarth et al. reported the transition‐metal‐free postpolymerization xanthylation of branched polyolefins (Figure 9b).^[219]^ This amidyl radical‐mediated C−H functionalization obviated the need for metal catalysts and was also shown to be chemoselective, regioselective, and scalable. In the following year, the Leibfarth group further unveiled an organocatalytic C−H fluoroalkylation strategy to functionalize aromatic commodity polymers.^[220]^ Specifically, they demonstrated that CF_3_ moieties could be installed on the pendant rings of PS when the polymer was subjected to trifluoroacetic anhydride and an organic photoredox catalyst in the presence of blue light (Figure 9c). Of the phenoxazine, phenothiazine, and phenazine photocatalysts that were screened, the phenazine‐based catalyst was found to be optimal in their case. Notably, these organocatalysts possess the requisite reduction potentials to replace ruthenium‐based catalysts in the photocatalytic cycle. However, the relatively short excited‐state lifetimes of organic photoredox catalysts still pose an outstanding challenge and so there remains much room for further exploration and advances in this area.

Looking ahead, we expect that C−H functionalization, i.e., both C(sp^3^)−H and C(sp^2^)−H, will continue to be an interesting avenue for further exploration in the area of polymer upcycling. As many of the hitherto published methods have necessitated the use of potentially toxic and expensive transition‐metal catalysts, future work in this area will likely lean towards employing noble‐metal‐free C−H activation strategies. Such is the aforementioned work by Leibfarth et al. involving the trifluoromethylation of PS,^[220]^ which could serve as an ideal starting point for subsequent development. Given the facile tunability of organic photoredox catalysts and the rapid developments from this subfield of catalysis, one can expect the scope of organocatalyzed, light‐driven C−H functionalization to expand significantly in the coming years. Aside from organic photoredox catalysis, there are also other potentially metal‐free approaches that may be investigated in the context of polymer upcycling. These could include organocatalyzed C−H functionalization methods,[^221^, ^222^, ^223^] aerobic C−H functionalization strategies that use molecular O_2_ as a sustainable oxidant in place of metal catalysts,^[224]^ metal‐free electrochemical oxidative C−H trifluoroalkylations,[^225^, ^226^, ^227^] and biocatalytic methods that employ artificial enzymes for direct and selective C−H functionalization.[^228^, ^229^] This list is not exhaustive and many new methodologies emerging from the field of organic synthesis can conceivably be applied to polymer upcycling, though the efficacy of such reactions on polymers will remain an open question until further investigations can be carried out.

### 2.10 Selective Chemical Recycling

Notwithstanding the environmental crisis brought about by plastics pollution, the equilibrium between the production of commodity plastics and their recycling options remains imbalanced. Mechanical and chemical recycling appear to be the most promising approaches for recycling plastic waste, since incinerating or dumping them in a landfill simply results in air/land pollution. The lack of economic incentives and technologies for the generation of high‐value‐added products via mechanical recycling^[230]^ and the inability to effectively recycle many plastics, have prompted the research community to develop efficient catalyst systems for chemical recycling, i.e., tertiary recycling. This method involves depolymerizing plastics into monomers or oligomers, which are then repolymerized to yield recycled polymer with the original materials properties (closed‐loop recycling) or to yield a new material dissimilar to the virgin plastic (open‐loop recycling). This approach of using catalysis in a rapid and selective manner has great potential in paving the way towards a circular plastics economy.^[231]^ Nevertheless, it is often more energy intensive and expensive to implement, in comparison to mechanical recycling and incineration.^[232]^ Moreover, the heterogeneity and wide range of compositions in plastic waste streams make the chemical recycling approach difficult to implement.^[233]^ Indeed, it is not trivial to find efficient, sustainable, and adaptable methodologies to selectively depolymerize mixtures of different polymers in the presence of additives, dyes, pigments, fillers, antioxidants, and/or plasticizers.

**Figure 10:**
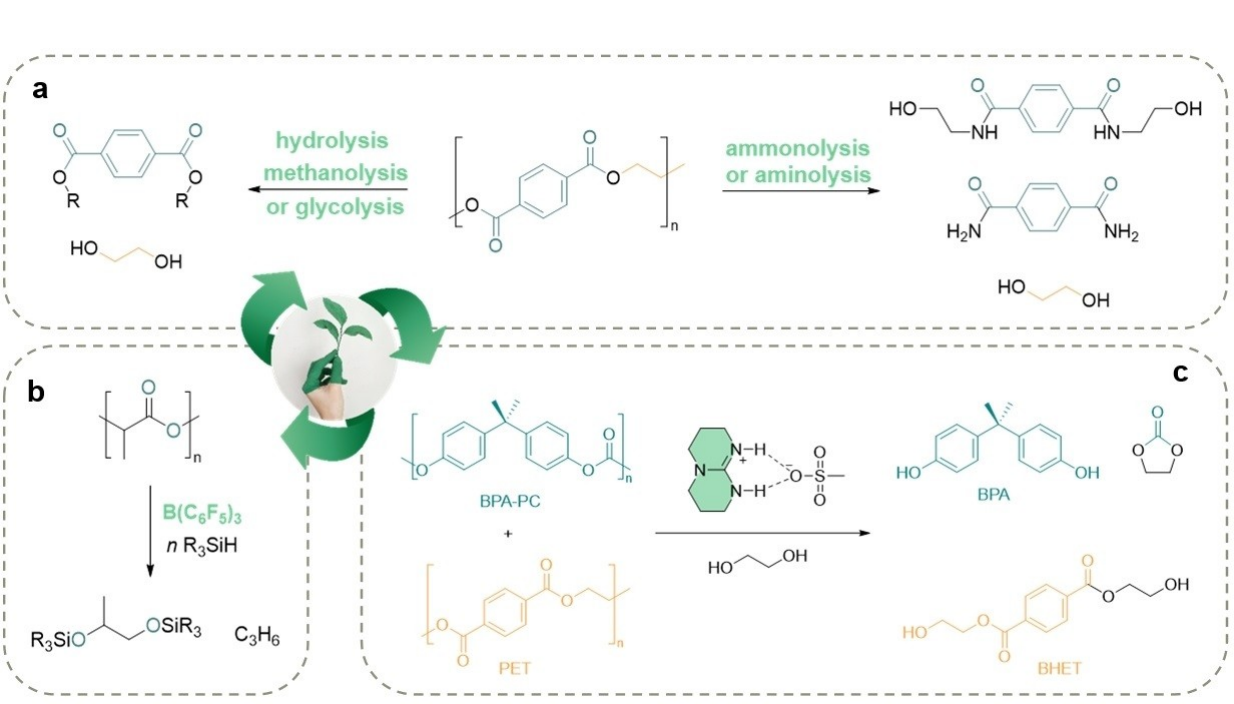
a) PET chemical recycling routes by using organic catalysts. b) PLA depolymerization promoted by B(C_6_F_5_)_3_.^[234]^ c) Selective depolymerization of BPA‐PC and PET.^[150]^

While clearly the catalytic process depends on the chemical nature of the plastic to be transformed and potentially these catalysts could be designed to tackle a specific plastic, the majority of the depolymerization reactions are conducted in a nonselective fashion, affording a mixture of products difficult to separate industrially. For instance, several organocatalysts have been used for depolymerizing poly(ethylene terephthalate) (PET), the most commonly recycled plastic, but little attention has been paid to realplastic waste streams. While organic bases, ureas, or ILs[^235^, ^236^, ^237^] have served as catalysts for recycling PET (i.e., neutral hydrolysis, methanolysis, or aminolysis; Figure 10a) the use of these catalysts on real plastic streams is rare.^[119]^ A remarkable example was reported by Liu and co‐workers, who designed ILs containing protonated imidazole anions and DBU cations for the methanolysis of PET and PC materials at lower temperatures compared to previously discussed catalysts. This can pave the way to selective chemical depolymerization.^[238]^

However, a few examples have shown that depolymerizations via hydrogenolysis,^[239]^ hydrosilation,[^240^, ^241^] and alcoholysis^[242]^ could be performed in a selective manner. In 2015, Cantat et al. showed that real plastics streams based on polyethers, polyesters, and polycarbonates could be chemically treated by sequential depolymerization reactions under metal‐free conditions by using B(C_6_F_5_)_3_ or [Ph_3_C^+^,B(C_6_F_5_)^4−^] as organocatalysts (Figure 10b).^[234]^ More recently, it was shown that stable acid–base mixtures (e.g., TBD:MSA) could be also applicable to plastic waste mixtures based on PET and BPA‐PC^[150]^ and commercial blends that contain colorants and other additives (Figure 10c). This catalyst not only demonstrates that under suitable conditionscatalytic depolymerization could be performed in a selective fashion, but also that plastic waste could be used as an alternative feedstock for the synthesis of high‐value‐added molecules.

In conclusion, inadequate infrastructure and technologies for plastic sorting, together with limited systems to selectively tackle a given plastic in the presence of additives, colorants, or other plastics currently limits the full potential of organocatalytic chemical recycling approaches. While it is true that future solutions will likely be more focused on the development of biodegradable materials or completely recyclable polymers, legacy plastics will still be one of the major components of plastic waste streams and selective routes for chemical depolymerization involving organocatalysts maximize the plastic life cycle. For this reason, catalyst design has to focus on a few key concepts: 1) efficiency of depolymerization, 2) high selectivity for specific products that are easily separated from impurities, 3) ability to use low catalytic loadings without compromising polymer molecular weights, and 4) preferably robust (thermally stable) and reusable catalysts.

## 3 Summary and Outlook

Transition‐metal‐free catalysts comprise a large array of compounds established among the preferred methods for promoting chemical transformations. Over the past few years, transition‐metal‐free catalysts have also emerged in several polymerization and depolymerization reactions owing to their greener character, higher selectivity, tunable versatility, and easier removal in comparison to metal catalysts. Indeed, the exponential growth in the number of articles and impact over the past 20 years highlights the tremendous academic interest in the field. The nascent work in organo‐ and transition‐metal‐free catalyzed reactions demonstrates the wealth of opportunities for transforming the fields of controlled radical polymerization, additive manufacturing, and circular economy of plastics.

In this Review, we have highlighted 10 nascent fields where we believe that organocatalysis has not yet reached its full potential. These organocatalysts and transition‐metal‐free promoters will undeniably bring to humankind future innovations that will lead to a plethora of previously inaccessible reaction pathways. It must be noted that though not covered in this Review, several remarkable contributions have been published recently regarding the use of transition‐metal‐free catalysts for ring‐opening metathesis polymerization (MF‐ROMP).[^80^, ^243^] In this regard, pyrylium salts have been incorporated as effective photocatalysts in combination with an electron‐rich vinyl ether to initiate the process. This approach delivered excellent temporal control over the polymerization reaction, although with considerably lower functional group tolerance than conventional metal‐catalyzed ROMP.^[244]^ Nevertheless, MF‐ROMP has been demonstrated to be effective for the preparation of block copolymers in combination with ROP and a bifunctional initiator^[245]^ and to perform stereoselective ROMP.^[246]^ Although in Section 2.1 we discussed the contribution of organocatalysis mediation in O‐ATRP, other important contributions have been made in living radical polymerization. We would especially like to highlight the work by Goto and co‐workers, whereby controlled living radical polymerization of a variety of chemically distinct monomers was achieved via an organocatalyzed process involving halogen exchange.^[247]^

We strongly believe that while there are a great number of organocatalyst families, the design and implementation of de novo organocatalysts would be needed to facilitate faster progress in these fields. These de novo designs must be achieved after proper elucidation of the catalyst role in (de)polymerization reactions by expanding the reaction scope and complementing them with in situ operando spectroscopy characterization techniques and density functional theory tools.

Benchmarking the current state of the field, it is clear that despite the demonstrated benefits of transition‐metal‐free catalysts, they still lag far behind the metal‐based catalysts. Moreover, care must be taken when claiming that organocatalysts are nontoxic, as some reports have shown that in certain cases, they are indeed cytotoxic.^[7]^ In particular, future innovations in organocatalysis should focus on solving problem arising from high catalyst loading, poor thermal stability, low light absorption, lack of chemical selectivity, and the absence of reliable cross‐coupling and stereocontrolled reactions. In short, numerous opportunities present themselves in these burgeoning fields, and we expect many more exciting discoveries in the next few years.

## Conflict of interest

The authors declare no conflict of interest.

4

## Biographical Information

*Dr. María Valle earned her BSc in Chemistry and her Master in Synthetic and Industrial Chemistry from the University of Valladolid (Spain). In 2015, she started her PhD at the same university under the supervision of Prof. Rafael Pedrosa and Prof. José María Andrés. There, she developed new homogeneous and supported chiral bifunctional catalysts (thioureas and squaramides) for the synthesis of diastereo‐ and enantiomerically pure compounds. Later, she conducted postdoctoral research at the Institute of Molecular and Translational Medicine in Olomouc (Czech Republic), where she spent two years working on the synthesis of new molecules that allow the simultaneous detection of different enzymes Since November 2020, she has been working as a postdoc at POLYMAT in the group of Dr. Haritz Sardon*.

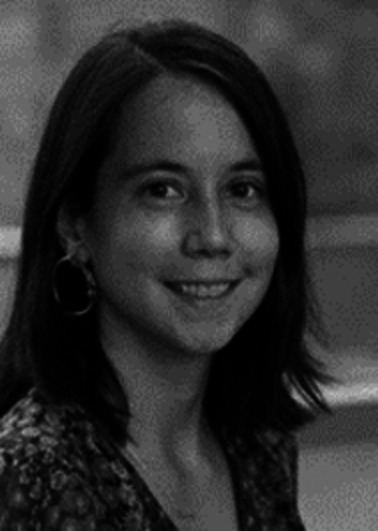

## Biographical Information

*Dr. Marta Ximenis earned her BSc in Chemistry from the University of the Balearic Islands (Spain) in 2013 and her PhD in 2019 under the supervision of Prof. Costa. During this time, she developed squaramide‐based self‐immolative spacers for drug delivery applications. In 2020 she moved to Japan and joined Prof. Uemura's laboratories at the University of Tokyo. She worked there for two years on the synthesis of 2D polymer networks using metal–organic frameworks as template nanoreactors. Since January 2022, she has been a postdoctoral fellow at POLYMAT in the group of Dr. Haritz Sardon. Her research interest focuses on the chemical recycling of commodity plastics using organocatalysis*.

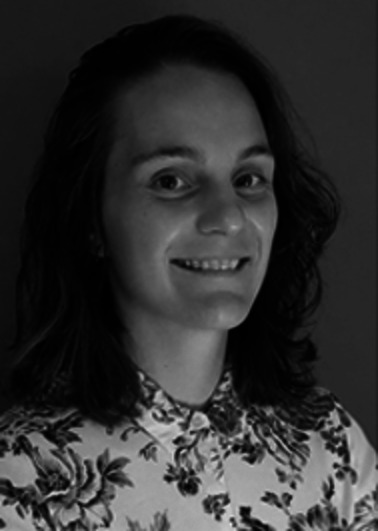

## Biographical Information

*Xabier Lopez de Pariza is currently a PhD candidate under the supervision of Dr. Haritz Sardon at POLYMAT Institute, University of the Basque Country. He received his BSc in Chemistry in 2018 and MSc in Chemistry and Polymers in 2019 both at the University of the Basque Country. During that period, he worked on the continuous‐flow polymerization of polyurethanes under the supervision of Dr. James L. Hedrick at IBM‐Almaden Research Center (San Jose, California). His current research interests include the design and study of organic photocatalysts for sustainable additive manufacturing applications*.

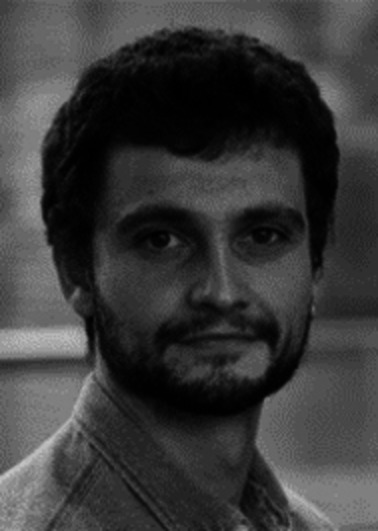

## Biographical Information

Dr. Julian Chan is a Scientist III (PI) and Manager (Talent Development) at A*STAR, Singapore. He received his B.S. in Chemistry from University of Illinois at Urbana‐Champaign (USA) in 2005 and his PhD degree in Organic Chemistry from Massachusetts Institute of Technology in 2010. After postdoctoral stints at University of California, Berkeley (2010–2012) and IBM Almaden Research Center (2012–2014), Dr. Chan took up a position as Assistant Professor at the University of Ottawa (Canada) from 2015 to 2020. In 2021, he moved to Singapore and joined the Institute of Sustainability for Chemicals, Energy and Environment (ISCE^2^) at A*STAR. His portfolio includes functional π‐conjugated organic materials, polymers for nanomedicine, and sustainable polymers.

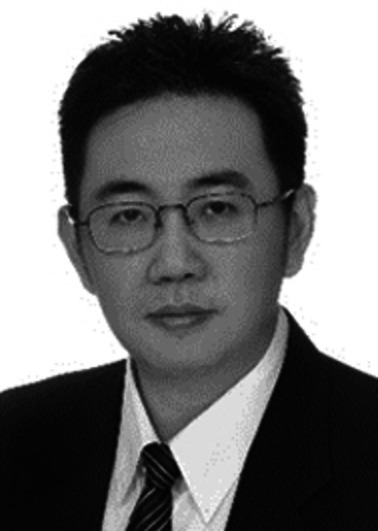

## Biographical Information

*Dr. Haritz Sardon received his PhD in 2011 at the University of Basque Country under the supervision of Prof. Lourdes Irusta and Prof. M. J. Fernandez‐Berridi before joining the group of Dr. James Hedrick at IBM‐Almaden Research Center (USA) as a postdoc. In 2014, Haritz returned to Spain with a Ministry grant and joined POLYMAT institute as junior group leader before starting his independent research career as an associate professor at the University of Basque Country in 2017. His work is focused on organocatalysis for polymerization/depolymerization reactions, including step‐growth polymerization of polyurethanes, polycarbonates, polyesters and polyethers as well as depolymerization of commodity plastics*.

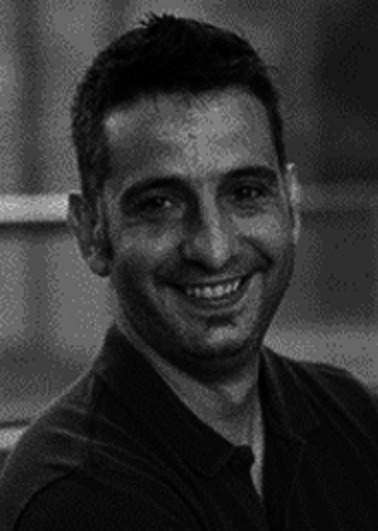

## Data Availability

Data sharing is not applicable to this article as no new data were created or analyzed in this study.
